# Old Sonic Hedgehog, new tricks: a new paradigm in thoracic malignancies

**DOI:** 10.18632/oncotarget.24411

**Published:** 2018-02-06

**Authors:** Etienne Giroux Leprieur, David M. Jablons, Biao He

**Affiliations:** ^1^ Department of Respiratory Diseases and Thoracic Oncology, APHP - Ambroise Paré Hospital, Boulogne-Billancourt, France; ^2^ EA 4340 BCOH, UVSQ, Paris-Saclay University, Boulogne-Billancourt, France; ^3^ Thoracic Oncology Program, Department of Surgery, Helen Diller Family Comprehensive Cancer Center, University of California San Francisco, San Francisco, California, USA

**Keywords:** Sonic Hedgehog, lung cancer, mesothelioma, cancer stem cell, chemoresistance

## Abstract

The Sonic Hedgehog (Shh) pathway is physiologically involved during embryogenesis, but is also activated in several diseases, including solid cancers. Previous studies have demonstrated that the Shh pathway is involved in oncogenesis, tumor progression and chemoresistance in lung cancer and mesothelioma. The Shh pathway is also closely associated with epithelial-mesenchymal transition and cancer stem cells. Recent findings have revealed that a small proportion of lung cancer cells expressed an abnormal full-length Shh protein, associated with cancer stem cell features. In this paper, we review the role of the Shh pathway in thoracic cancers (small cell lung cancer, non-small cell lung cancer, and mesothelioma) and discuss the new perspectives of cancer research highlighted by the recent data of the literature.

## INTRODUCTION

The Sonic Hedgehog (Shh) signaling pathway is physiologically activated during embryogenesis and development [1]. The hedgehog gene was originally discovered in Drosophila in 1980 and described by Nüsslein-Volhard and Wieschaus [2]. The name “hedgehog” comes from the spiky aspect of the embryonic cuticle observed in the mutant for this gene. In mammals, the Shh pathway plays a major role in development of the brain, the limbs, and pulmonary organogenesis. The Shh pathway is also reactivated in various solid cancers, such as thoracic cancers including small-cell lung cancer (SCLC) [3], non-small cell lung cancer (NSCLC) [4–6] and malignant pleural mesothelioma (MPM) [7]. Shh activation is associated with the maintenance of cancer stem cells (CSCs) and is involved in early stages of carcinogenesis [8, 9]. In addition, the Shh pathway is associated with resistance to radiotherapy and chemotherapy in several models of cancer. Until recently, however, the role of the Shh pathway in thoracic cancers was poorly described. Recent studies have expanded our comprehension of the role of Shh pathway activation and its interaction with CSCs in thoracic cancers.

In this review, we aim to describe the current knowledge of CSCs in lung cancer and detail the mechanisms of activation of the Shh pathway in thoracic malignancies (SCLC, NSCLC, and MPM), with a focus on recent discoveries of the correlation between Shh expression and CSCs in lung cancer.

## GENERALITIES OF CSCS IN LUNG CANCER

CSCs represent a very small proportion of tumor cells and are undifferentiated, with high oncogenesis, proliferation and differentiation potential. These cells are involved at the very early steps of oncogenesis. CSCs are defined by three essential properties: self-renewal, production of differentiated “daughter” cell lines and *in vivo* oncogenic capacity (xenograft formation) [10]. Several key genes are expressed in CSCs, such as *SOX2*, *NANOG* and *OCT4/POU5F1.* CSCs have been found in many solid tumors, including lung cancer. Carney et al. showed that some NSCLC and SCLC cells, representing less than 2% of tumor cells, were able to produce new tumor cell colonies. These colonies could induce tumors *in vivo* similar to the initial tumor in nude mice [11].

Several different biomarkers have been described so far for the detection and isolation of CSCs. Membranous markers are the most commonly used for CSCs detection, such as CD133, CD44, and breast cancer resistance protein (BCRP). CD133 is a transmembrane receptor with unknown function. CD133+ cells represent between 0.30% and 6% of tumor cells in NSCLC and exhibit the ability to grow as spheroids in agarose culture and to differentiate to CD133- tumor cells, and show high *in vivo* oncogenic potential in *NOD/SCID* mice [12, 13]. CD44 is a membranous protein involved in cell adhesion, migration and interaction with the extracellular matrix [14]. Notably, CD44 interacts with EGFR and Met [15, 16]. In NSCLC, CSC genes (*NANOG, OCT4/POU5F1,* and *SOX2*) are overexpressed in CD44+ cells [17]. BCRP is a member of the ABC transporter family that is also overexpressed in CSCs and involved in chemoresistance [18, 19]. The phenomenon of Hoechst 33342 efflux (“side population” (SP)), i.e. the active transport of Hoechst 33342 from the intracellular compartment to the extracellular space through membranous ABC transporters, has also been used as a marker of CSCs [18]. More recently, several studies have validated ALDH (aldehyde dehydrogenase) activity as a marker of CSC, especially in NSCLC [20, 21]. ALDH represents a group of enzymes responsible for the oxidation of aldehydes. Their activity appears to be increased in stem cells. ALDH activity is involved in alcohol metabolism, vitamin A metabolism, resistance to certain chemotherapy agents (such as cyclophosphamide) and early differentiation of stem cells [22]. In NSCLC, ALDH+ cells display a CSC phenotype, both *in vitro* and *in vivo* [20, 21]. Specific techniques using ALDH activity that can be used in NSCLC have been developed [20, 21], such as ALDEFLUOR™ (StemCell Technology) [23].

Despite the identification of CSC markers, several issues remain, limiting the potential use of such CSC markers in lung cancer. Meng et al. showed that CD133 was not specific enough to isolate CSCs *in vitro* in NSCLC cell lines, as some CD133- cells had CSC features (colony formation, self-renewal, proliferation, differentiation, and chemoresistance) [24]. Moreover, PCR and flow cytometry analyses in lung cancer cell lines showed that CD133 was much more specific to CSCs in SCLC than in NSCLC [25]. Finally, the interpretation of ALDH activity can be problematic in lung tumors, as it can also be increased in normal pneumocytes from smokers [26]. The use of several markers to reach good specificity for CSC isolation in flow cytometry has also been described [27], but this increases the complexity of the assays and requires the use of several lasers with strict rules of compensation, inducing a possible higher rate of false-positives and false-negatives. New biomarkers for CSCs detection and isolation are therefore needed.

## THE SHH PATHWAY

### The Shh protein

Three different types of hedgehog proteins have been described: Indian, Desert and Sonic Hedghehog (Shh). In mammals, the Shh protein is the main expressed protein. In physiological conditions, the *SHH* gene, located in q736, produces a pre-protein of 45 kDa. The pre-protein contains an auto-cleavage site, and cleavage results in the production of a 20 kDa N-terminal protein (Shh-N) and a 25 kDa C-terminal protein (Shh-C) [28]. The Shh-C protein has cholesterol transferase activity and catalyzes the addition of cholesterol to the C-terminus part of the Shh-N protein [29, 30]. In addition, palmitoyl residues are also covalently attached to the Shh-N protein (palmitoylation). These lipidic modifications enable the Shh-N protein to anchor to the cell membrane before being secreted into the extracellular space [31]. All physiological functions during development are linked to the Shh-N protein, and the palmitoylated Shh-N protein is approximately 30 times more active than its non-palmitoylated form [32]. The Shh-C protein has no known physiological role outside its cholesterol transferase activity and is also freely secreted into the extracellular space.

### Activation of the Shh pathway

**Figure 1:**
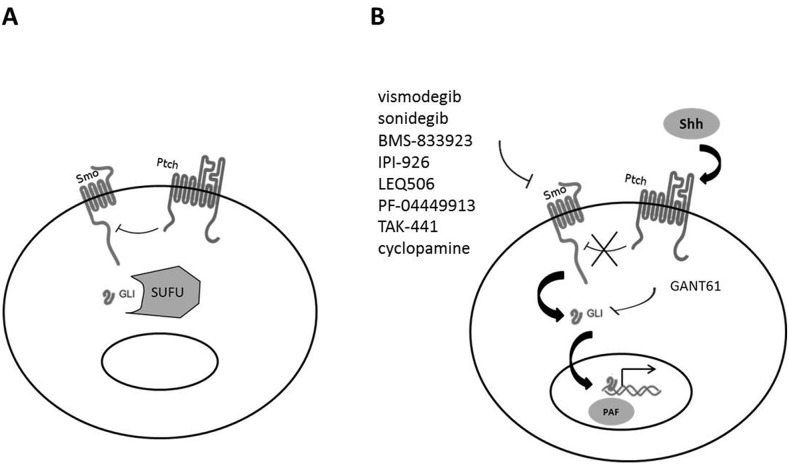
Shh signaling pathway **(A)** In the absence of Shh binding to Ptch, Ptch exerts an inhibitory action on Smo. Gli is associated with SUFU in the cytoplasm and is inactive. **(B)** When Shh binds to Ptch, Smo is activated and induces the migration of Gli into the nucleus with subsequent activation of the transcription of target genes. Main inhibitors of the Shh pathway are also shown.

The receptor for Shh is Patched (Ptch), a 12-transmembrane domain receptor, with two isoforms (Ptch1 and Ptch2). In the absence of ligand (Figure 1A), Ptch inhibits the migration of Smoothened (Smo) to the membrane, keeping Smo inactive inside the cell. The Gli transcription factors (Gli1, Gli2 and Gli3) are bound to the protein SUFU (suppressor of fused homolog) and remain inactive in the cytoplasm [33]. Gli3 is also phosphorylated by PKA (protein kinase A), which induces the formation of a GLIR inhibitor protein that represses the transcription of the Shh-target genes.

When Shh binds to its receptor Ptch (Figure 1B), Smo migrates to the cell membrane. Gli proteins then dissociate from SUFU and translocate into the nucleus to activate the transcription of target genes.

Several mechanisms that negatively regulate the Shh pathway under physiological conditions have been described. HHIP (hedgehog interacting protein) binds to Shh on the cell membrane and thus competes with Ptch [34]. Upon Shh binding to Ptch, the Shh-Ptch complex is internalized in the cell before its degradation by the lysosome, stopping the activation of Smo [35]. The ZIC transcription factors interact with Gli proteins through their zinc finger domain [36] and either activate or inhibit Gli, depending on the cell type and physiological conditions [37].

## SHH PATHWAY AND SOLID CANCERS

### Mechanisms of activation of the Shh pathway in solid cancers

Several mechanisms have been described for the reactivation of the Shh pathway in solid cancers. The Shh pathway can be activated by somatic mutations. For example, Gorlin syndrome, which induces the formation of multiple basal cell carcinomas, rhabdomyosarcomas and medulloblastomas, is characterized by the presence of inactivating mutations of *PTCH* that result in constant activation of Smo [38, 39]. *SUFU* mutations have also been described in sporadic cases of medulloblastomas [40]. In sporadic basal cell carcinomas, approximately 70% of cases present a mutation of Shh pathway-related genes (inactivating mutation of *PTCH* or activating mutation of *SMO*) [41]. The importance of the activation of the Shh pathway in this type of cancer has led to the development of a Smo inhibitor (vismodegib, Genentech, USA) for the treatment of basal cell carcinoma [42].

In several other cancers, an activation of the Shh pathway was described without evidence of mutation of Shh pathway-related genes. The mechanism of activation is autocrine and paracrine activation through the secretion of the Shh protein. This mechanism of activation has been described in breast [43–45], hepatic [46, 47], pancreatic [48], gallbladder [49], gastric [50] and prostatic cancers [51, 52]. A recent meta-analysis on 39 studies (4496 cases) showed a prognostic role for Gli1 expression in most solid tumors, with worse 3-year, 5-year, and 10-year overall survival and disease-free survival in case of Shh pathway activation [53].

In cancer cells, various genes are activated by Gli proteins, depending on the context and the type of cell [54]. Most of the Gli target genes are involved in proliferation, cell survival, epithelial-mesenchymal transition (EMT) and CSC phenotype. Some genes are also involved in negative control of the Shh pathway. Table 1 summarizes the main target genes of the Shh pathway in cancer.

**Table 1:** Main cellular processes and genes activated by the Shh pathway

Cellular processes	Gene
	*FOXC2*
	*SNAI1*
**EMT**	*TWIST2*
	*ZEB1*
	*ZEB2*
	*JAG2*
	*FST*
	*GREM1*
	*BMP4*
	*WNT2B*
**CSCs**	*WNT5A*
	*PDGFRA*
	*BMI1*
	*LGR5*
	*CD44*
	*CD133*
**Positive feedback of the Shh pathway**	*GLI1*
**Negative feedback of the Shh pathway**	*PTCH1*
	*PTCH2*
	*HHIP1*
**Cell proliferation**	*MYCN*
	*CCND1*
	*CCND2*
	*NEAC*
	*FOXM1*
	*CCNB1*
	*CDC25B*
**Cell survival**	*BCL2*
	*CFLAR*
**Other processes**	*FOXF1*
	*FOXL1*
	*PRDM1*
	*PTHLH*

Adapted from [55].

EMT: epithelial-mesenchymal transition; CSCs: cancer stem cells.

### The Shh pathway and CSCs

The relationship between CSCs and the Shh pathway has been well documented. Li et al. isolated cells with a CSC phenotype (CD44 (+) CD24 (+) ESA (+)) from primary cultures of pancreatic adenocarcinomas [8]. These cells had a 100-fold greater tumorigenesis potential than other cells, as measured by the formation of xenografts in immunocompromised mice. These cells also showed overexpression of Shh pathway compared with other tumor cells. In another work on lung cancer cell lines (HCC and H1339), inhibition of the Shh pathway by a pharmacological Smo-inhibitor (vismodegib) resulted in a decrease of the population of CSCs measured by the ability of efflux of Hoechst 33342 (SP+ cells) using flow cytometry analysis [55]. The population of SP+ cells decreased from 0.45% (HCC) and 0.75% (H13339) to 0.24% (HCC) and 0.18% (H1339) after treatment with vismodegib. Similar results were shown in PTEN-dependent glioblastoma models [56]. In gallbladder cancer, CSCs isolated by Fluorescence-activated cell sorting (FACS) (through CD44 and aberrantly glycosylated integrin α3β1 staining) showed high level of Shh pathway activation, which was required for CSC renewal [57]. Finally, Lemjabbar-Alaoui et al. used a model of lung carcinogenesis induced by cigarette smoke in which human bronchial cells from primary cultures were exposed to tobacco smoke for 8 days [9]. After 8 days of tobacco exposure, bronchial cells had acquired a tumor phenotype, with an increase of proliferation rate, capacity of growth on agarose gel, and ability to form tumor xenografts in immunocompromised mice. The authors also found an activation of the Wnt and Shh signaling pathways in these cells. Inhibition of the Shh pathway prevented the tobacco-induced tumor phenotype. Interestingly, nicotine seems to induce the proliferation of CSCs in pancreatic cancer through the activation of the Shh pathway [58].

Several factors of cell proliferation are overexpressed in CSCs, such as Myc proteins [59]. A correlation between the Shh pathway and Myc activation has been shown in several studies [60–62]. Gli inhibition by GANT-61 inhibits CSC proliferation *in vitro* and *in vivo* and the expression of c-Myc and CSC genes (*NANOG, OCT4,* and *SOX2*) [63]. In NSCLC, c-Myc activation is closely associated with hPAF1C, a transcription regulator activated in CSCs [64, 65]. hPAF1C expression has prognostic impact in early-stage NSCLC and showed a correlation with c-Myc expression in tumor samples and in NSCLC cell lines [66]. Interestingly, the inhibition of hPAF1C also inhibits Shh expression [65].

### Activation of the Shh pathway in NSCLC

Several studies have demonstrated that the Shh pathway is activated in NSCLC. Yuan et al. showed an activation of the Shh pathway in a number of NSCLC cell lines [4]. The authors also analyzed the expression of Gli1 by immunohistochemistry in a panel of 120 samples of NSCLC in a tissue microarray and found that 87% of adenocarcinomas and 93% of squamous cell carcinoma demonstrated Shh pathway activation. Another study performed immunohistochemistry analyses of several components of the Shh pathway on 80 surgical samples of NSCLC (stage I to III); a large majority of the samples were positive for Shh (97.5%), Ptch1 (78.7%), Smo (72.5%), Gli1 (98.7%) and Gli2 (87.5%) [5]. The expression of Shh pathway factors was present only in tumor cells and not in normal tissues. In another study of 248 early-stage NSCLC, the expression of Shh pathway factors was not correlated to recurrence-free survival or overall survival [6].

The pro-oncogenic role of the Shh pathway in NSCLC was confirmed by Huang et al. [67]. The authors analyzed the expression of Shh pathway genes by PCR in two different cohorts of patients with lung squamous cell carcinoma (n = 178 and n = 56), as well as in four squamous cell carcinoma cell lines (H520, H2170, H226, and SK-MES-1). The results showed that the Shh pathway was activated both in the cell lines and in the two cohorts. *In vitro*, inhibition of the Shh pathway by Gli2 siRNA or a Gli-inhibitor molecule (GANT61) decreased cell proliferation and activated apoptosis. The use of GANT61 in xenograft models *in vivo* also showed antitumor activity.

### Shh and chemoresistance in NSCLC

Concerning the role of the Shh pathway in chemoresistance, a correlation has been shown between the activation of the Shh pathway in cancer cells and the expression of ABC (ATP-binding cassette) transporters, such as MDR1 and ABCG2, which have demonstrated involvement in chemoresistance [68]. We showed that activation of the Shh pathway was correlated with the acquisition of a mesenchymal phenotype (EMT) by tumor cells, a known factor of chemoresistance, with an inverse correlation between the expression of Gli and epithelial markers such as E-cadherin and a positive correlation with migration and invasion [69, 70]. Moreover, vismodegib exhibits anti-tumor activity in cisplatin-resistant cancer cell lines [71]. Ahmad et al. performed analyses using the A549M NSCLC cell line, in which A549 cells were made resistant to cisplatin and erlotinib by treatment with TGFβ and acquisition of a mesenchymal phenotype [72]. The IC50 values of cisplatin and erlotinib in A549M cells were significantly lower with pretreatment by vismodegib or anti-Shh siRNA. Moreover, the administration of vismodegib decreased the expression of CSC-related genes, such as *SOX2* and *NANOG*. Finally, we demonstrated that chemorefractory NSCLC (*i.e.* NSCLC with early tumor progression with first-line platinum-based chemotherapy) showed overexpression of Gli2 compared with chemosensitive NSCLC and that inhibition of Shh pathway had a synergistic effect with cisplatin in the most chemoresistant cell lines *in vitro* [73].

### Shh and resistance to EGFR tyrosine kinase inhibitors in EGFR-mutated NSCLC

During embryogenesis, there is a cross-talk between the Shh and EGFR pathways [74]. Kim et al. showed a correlation between the expression of Shh-related proteins and the presence of EGFR mutation in surgically resected lung adenocarcinoma [75]. In *EGFR*-mutated NSCLC, pre-clinical models have suggested a role of the Shh pathway activation in tumor progression with EGFR tyrosine kinase inhibitors (TKIs). The Shh pathway is closely associated with EMT, as discussed above, and EMT is one of the mechanisms associated with acquired resistance to EGFR TKIs [76]. In addition, *EGFR*-mutated lung cancer cells resistant to EGFR TKIs also harbor *SMO* amplification along with Met activation [77]. In these cells, combined Shh and Met inhibition had significant antitumor activity. The potentiation of the activity of EGFR TKIs by the inhibition of the Shh pathway has been proven in several other pre-clinical studies, with an inhibition of CSC renewal and activity [78–80]. The identification of drugs targeting both Met and Smo could lead to the development of new treatments for *EGFR*-mutated NSCLC [81].

### Shh and resistance to radiotherapy

Activation of the Shh pathway has also been correlated with resistance to radiotherapy. Radiation treatment in hepatocellular carcinoma, colorectal and pancreatic carcinoma induced activation of the Shh pathway *in vitro* [47, 82]. Shh pathway activation is also associated with the risk of local tumor relapse in cervical carcinoma after radiotherapy [83]. *In vitro* irradiation of NSCLC cell lines induced overexpression of CSC markers, as CD44 and CD166, and also EMT [84].

### Activation of the Shh pathway in SCLC

Watkins et al. demonstrated that the Shh pathway was activated in SCLC, with an autocrine and juxtacrine activation mechanism [3]. Immunohistochemistry and western blot analysis of SCLC human samples and cell lines showed an overexpression of Shh and Gli1 in tumor and cancer cells. Inhibition of the Shh pathway by cyclopamine (a Smo inhibitor) blocked cell growth *in vitro* and *in vivo* in SCLC xenografts. These results were confirmed in transgenic mouse models with deletion of *RB1* (*retinoblastoma 1*) and *TRP53* (*transformation related protein 53*) [85]. Tumor growth was inhibited by deletion of Smo in these models and treatment with pharmacological inhibitors of the Shh pathway.

Smo inhibitors have been tested in clinical trials in SCLC. A phase II trial (Eastern Cooperative Oncology Group ECOG-1508 trial) evaluated the benefit of adding vismodegib to cisplatin and etoposide chemotherapy in advanced SCLC as first-line treatment [86]. No difference in terms of progression-free survival or overall survival was shown between patients who received only chemotherapy and those treated by vismodegib and chemotherapy. Several hypotheses could explain these negative results: no selection of a specific patient phenotype (inhibition of the Shh pathway benefits only non-responder tumors?), the absence of a predefined biomarker to identify patients who will likely benefit from vismodegib, or the treatment strategy used in this trial (concomitant treatment). A recent phase I trial testing another Smo antagonist (sonidegib) in addition to cisplatin-etoposide for advanced SCLC (n=15) showed a 79% response rate [87]. Interestingly, one patient experienced a prolonged progression free survival (27 months), with a tumor showing amplification of *SOX2*, a CSC-related gene. Finally, recent data suggested a complex mechanism of activation of the Shh pathway in SCLC. As the canonical Shh pathway (through Ptch-Smo activation) seems to be strongly involved in SCLC cancer cells, a non-canonical Shh pathway involving a Smo-independent upregulation of cyclin B1 induced chromosomal instability in cells lacking both p53 and Rb1 [88]. These Smo-independent mechanisms in SCLC could partially explain the negative results of Smo inhibitors in SCLC.

### Activation of the Shh pathway in MPM

Similar to lung cancer, previous studies have shown an overexpression of Shh pathway proteins in MPM, while normal pleural tissue does not express these proteins [7]. Activation of the Shh pathway in MPM seems to be regulated by the protein kinase CK2α, which is overexpressed in MPM [89]. Moreover, in models of primary cultures of MPM, Smo inhibition induced a significant decrease in tumor growth through Gli1 inhibition. The inhibition of Smo also had an anti-tumor action in animal xenograft models *in vivo*. Similar results were obtained by direct inhibition of Gli by siRNA or a small inhibitory molecule of Gli (Gli-I) [90, 91] or GANT61 [92]. Interestingly, inhibition of Gli with a Gli-I molecule had a synergistic cytotoxic effect with pemetrexed in MPM lines [90].

## FULL-LENGTH SHH PROTEIN AS A NEW CSC MARKER AND FUTURE THERAPEUTIC STRATEGIES

**Figure 2:**
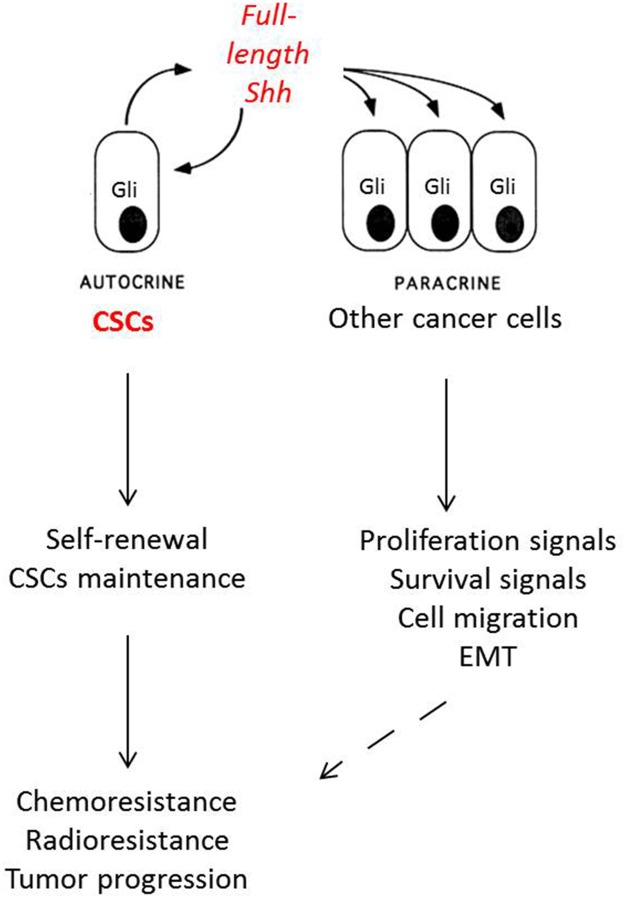
Mechanisms of Shh activation in non-small cell lung cancer Cancer stem cells (CSCs) are Shh+ cells that secrete Shh full-length protein and induce autocrine (self-renewal and CSC stock maintenance) and paracrine activity (proliferation and survival signals, cell migration and epithelial-mesenchymal transition). All these signals are responsible for resistance to chemotherapy, radiotherapy and tumor proliferation in non-small cell lung cancer.

While the Shh pathway is closely associated with CSCs, which represent less than 1% of tumor cells, the majority of NSCLC cells show positive Gli1 and Gli2 expression by immunohistochemistry [5, 69, 70], suggesting a complex mechanism of Shh pathway activation in NSCLC. The main hypothesis is that the CSCs are the source of Shh production in the tumor, with paracrine activity and activation of downstream Shh pathway proteins (Figure 2). We confirmed this hypothesis in a recent work [93]. We used FACS to identify a small proportion of Shh-positive (Shh+) cells. When analyzing *SHH* gene expression by digital droplet PCR on sorted cells, we found that only Shh+ cells expressed *SHH*, whereas Shh-negative (Shh-) cells had no significant *SHH* expression. These Shh+ cells had paracrine activity on Shh- cells in term of proliferation and migration, with activation of Ptch, Smo and Gli in Shh- cells. Moreover, the Shh+ cells harbored CSC features, with spheroid formation in serum-free medium conditions, chemoresistance, and tumor initiation in nude mice, even with inoculation with as low as 1,500 cells. Surprisingly, while the full-length Shh protein is normally cleaved in the cytosol to produce functional N-terminal peptide, we found that Shh+ cells produced a full-length Shh protein that migrated to the membrane before its secretion. The underlying mechanisms of the production of unprocessed Shh protein are not yet clearly understood, and further studies are in process to clarify these mechanisms. Interestingly, the production of full-length Shh protein by CSCs does not seem to be restricted to NSCLC, as Shh+ can also be found in pancreatic adenocarcinoma, MPM and melanoma [93].

These findings of production of full-length Shh protein by CSCs represent a milestone in the understanding of CSC biology in lung cancer and implicate full-length Shh protein as a potential CSC marker. This new CSC marker, unlike many other markers used so far (CD133, CD44), has a functional role, and Shh-targeted therapies are already available. Treatment with vismodegib is able to induce a complete extinction of the Shh+ signal in FACS, both *in vitro* and *in vivo* [93]. Pre-treatment of NSCLC cells by vismodegib prevented tumor initiation in nude mice [93]. Targeting the Shh pathway should therefore be encouraged in association with chemotherapy to specifically inhibit CSCs and therefore enhance the efficacy of chemotherapy. One of the main challenges in targeting the Shh pathway is that all of the drugs currently available in clinical trials are Smo-inhibitors (shown in Table 2). But this strategy targets only the canonical Shh pathway, and non-canonical Shh pathway activation has been shown in various malignancies, such as in SCLC [88] or in MPM [91]. In these situations, targeting Gli could be more efficient, and the development of specific Gli-inhibitors is in process [91]. The other therapeutic option could be directly targeting the full-length Shh protein. We recently showed that treatment with an antibody specifically designed to target the C-terminal peptide of Shh protein, and therefore also the full-length Shh protein, showed encouraging anti-tumor activity *in vivo* [94], and could be a promising treatment in the future.

**Table 2:** Main Smo-inhibitors developed in clinical trials in thoracic oncology

Inhibitor	Company	Clinical development	Clinicaltrial ID	Type of thoracic malignancy
vismodegib (GDC-0449)	Genentech-Roche	Phase II	NCT02465060	SCLC
sonidegib (LDE225)	Novartis	Phase I	NCT01579929	SCLC
BMS-833923	Bristol-Meyers-Squibb	Phase I	NCT00927875	SCLC

SCLC: small cell lung cancer.

## CONCLUSIONS

The presence of Shh activation in thoracic cancers has been established for several years. However, the recent discoveries on the critical role of Shh as a CSC marker with a functional role and its impact on the global resistance to anti-cancer treatment and tumor proliferation have highlighted the pivotal role of this pathway in solid cancers, especially in NSCLC. Shh+ cells are the source of the full-length Shh protein production and exhibit CSC features, with a strong impact on oncogenesis and chemoresistance. A new avenue for the development of therapies targeting CSCs through Shh pathway inhibition is beginning, with expected improvements in terms of clinical benefits for patients with thoracic cancers.

BH and DJ reviewed the manuscript.
